# A Pilot Three-Dimensional Evaluation of Acetabular Bony Coverage After Modified Spitzy Shelf Acetabuloplasty

**DOI:** 10.3390/biomimetics11020117

**Published:** 2026-02-05

**Authors:** Fumito Kobayashi, Takehito Hananouchi, Kenichi Oe, Shohei Sogawa, Tomohisa Nakamura, Takanori Saito

**Affiliations:** 1 Department of Orthopaedic Surgery, Kansai Medical University, 2-5-1 Shinmachi, Hirakata 573-1010, Japan; kobayashi.fum@kmu.ac.jp (F.K.); kobebryant8913shohei@yahoo.co.jp (S.S.);; 2 Biodesign Division, Department of Academia-Government-Industry Collaboration, Office of Research and Academia-Government-Community Collaboration, Hiroshima University, 1-2-3 Kasumi, Minami-ku, Hiroshima 734-8551, Japan; 3 Translational Research Center, Hiroshima University, Hiroshima 734-8551, Japan; 4 Medical Engineering and Biodesign Laboratory, Graduate School of Biomedical and Health Sciences, Hiroshima University, Hiroshima 734-8551, Japan

**Keywords:** acetabular dysplasia, modified Spitzy shelf acetabuloplasty, three-dimensional analysis, computed tomography, bony coverage, 3D registration, acetabular sector Angle (ASA)

## Abstract

Modified Spitzy shelf acetabuloplasty is a joint-preserving surgical procedure for acetabular dysplasia that aims to enhance bony coverage of the hip joint. Although prior studies have primarily relied on two-dimensional (2D) radiographic evaluations, comprehensive three-dimensional (3D) assessments remain limited. The purpose of this retrospective study was to evaluate changes quantitatively in acetabular coverage following modified Spitzy shelf acetabuloplasty using 3D models reconstructed from computed tomography (CT) images. We retrospectively analyzed 11 hips in 11 patients who underwent staged bilateral modified Spitzy shelf acetabuloplasty. Preoperative and postoperative CT data were used to construct 3D pelvic models, which were registered using anatomical landmarks. Bone graft dimensions, insertion angle, and placement location were evaluated. Acetabular sector angles (ASA), representing circumferential coverage of the femoral head, were measured at 15° intervals on the functional pelvic plane and the anterior pelvic plane. The mean bone graft dimensions were 26.3 ± 3 mm (anteroposterior length) and 12.7 ± 2.7 mm (mediolateral length), providing coverage of 49.5° ± 9.1°. Postoperative ASA increased significantly from 34.5° to 60° on the functional pelvic plane and from 0° to 45° on the anterior pelvic plane (both *p* < 0.05). 3D analysis demonstrated that modified Spitzy shelf acetabuloplasty effectively enhanced anterosuperior acetabular bony coverage. Although this is a report of a few cases (11 hips), the above findings highlight the value of 3D evaluation in identifying postoperative changes that may not be detected using conventional 2D assessments. Also, further research analyzing the three-dimensional bone graft model revealed in this study may help inform the development of a more ideal biomimetic approach, not only in terms of shape but also function.

## 1. Introduction

Acetabular dysplasia of the hip is a major cause of osteoarthritis (OA) [1,2]. It is characterized by inadequate bony coverage of the femoral head, resulting in joint instability and increased mechanical stress on the acetabular rim, which leads to damage to the labrum and articular cartilage [3,4]. Joint-preserving surgical procedures for acetabular dysplasia, including pelvic osteotomies and shelf operations, are performed to enhance bony coverage and to normalize load transmission across the anterolateral acetabular rim [5,6]. Surgical techniques include Chiari osteotomy [7], transpositional osteotomy of the acetabulum [8], triple osteotomy [9], rotational acetabular osteotomy [6], periacetabular osteotomy [5], curved periacetabular osteotomy [10], spherical periacetabular osteotomy [11], and shelf operation [12] (Table 1).

Regarding the shelf operation which is a joint-preserving surgical procedure that enhances acetabular coverage by augmenting the acetabular rim with a bone graft, it was first described by König in 1891 [12], and numerous modifications, including those proposed by Albee [13], Spitzy [14], Lance [15], and others, have since been reported. Compared with reorientation pelvic osteotomies, the shelf procedure is less invasive and has minimal influence on the natural progression of the disease [16]. Previous studies have demonstrated improvements in two-dimensional (2D) radiographic parameters, including the Lateral Center Edge Angle (LCEA), Sharp angle, and Acetabular Head Index (AHI) [16,17,18,19]. An additional factor, the “height of the shelf,” defined as the vertical distance between the undersurface of the bone graft and the lateral margin of the acetabulum, has also been evaluated as an important indicator [17,19].

To the best of our knowledge, however, changes in bony coverage resulting from the shelf operation have not been evaluated using three-dimensional (3D) analysis. For example, when computed tomography (CT) is employed, the location of the bone graft in the anterosuperior region of the acetabulum should be assessed [20]. Furthermore, constructing 3D pelvic models from CT images enables evaluation of the spatial orientation of the graft relative to the native acetabulum.

The purpose of this study was to assess 3D changes in the acetabulum before and after the shelf operation using 3D models. Furthermore, we analyzed the LCEA and Acetabular Sector Angle (ASA) [21] in different planes and measured the size, placement position, and insertion angle of the bone graft.

## 2. Materials and Methods

### 2.1. Patient Selection

Between April 2006 and July 2021, 156 patients (174 hips) underwent modified Spitzy shelf acetabuloplasty for symptomatic acetabular dysplasia at our institution, representing the treated population during the study period. The surgical indication included hip pain associated with acetabular dysplasia, even in middle-aged patients and those with early osteoarthritic changes, with the aim of preserving the native joint and delaying total hip arthroplasty. No alternative pelvic osteotomy procedures for adult acetabular dysplasia were performed during this period.

**Figure 1:**
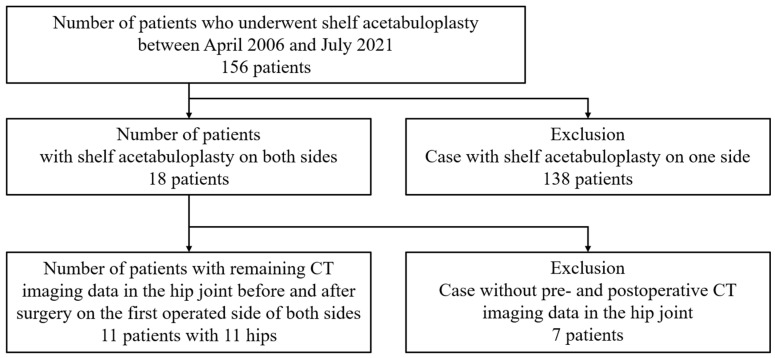
Patient selection. From the total of 156 cases, only those who underwent bilateral surgery were selected (18 cases). Among these, only cases with both preoperative and postoperative CT data were included (11 cases).

From this treated population, 138 patients who underwent unilateral surgery were excluded, leaving 18 patients who underwent staged bilateral procedures and were therefore eligible for three-dimensional analysis. Among these, 7 patients were further excluded due to the unavailability of both preoperative and postoperative CT data. Consequently, 11 patients (11 hips; 10 females and 1 male) were included in the final analysis, with one hip analyzed per patient. Preoperative and postoperative CT datasets (Revolution Frontier, GE HealthCare, Chicago, IL, USA) were obtained for three-dimensional reconstruction (Figure 1).

The mean age at the time of surgery was 30.2 ± 11.1 years (range: 16–45 years), and the mean body mass index was 22.7 ± 4.1 kg/m^2^ (range: 16.7–29.1 kg/m^2^). The mean interval between the first and second procedures was 1.3 years (range: 0.9–3.3 years), and the mean follow-up duration was 5.1 ± 2.7 years (range: 1.0–10.0 years). The OA stage was assessed using the Tönnis classification [21,22], with 8 hips classified as Grade 0 and 3 hips as Grade 1 (Table 2). This study was approved by Institutional Review Board of our institution (approval no. 2022171).

### 2.2. Surgical Procedure

All procedures were performed with the patient in the lateral position using the Smith–Petersen approach. A monocortical bone graft, approximately 35 mm in width, 30 mm in length, and 5 mm in thickness, was harvested from the iliac crest using a chisel. The lateral cortex of the ilium was exposed to the joint capsule through periosteal elevation, and the reflected head of the rectus femoris was detached from the acetabular rim. Under fluoroscopic guidance, two Kirschner wires were inserted along the joint capsule at the planned graft placement site. Using these wires as guides, a slot measuring 10 mm in depth and 3 mm in width was created. A curved chisel was then used to create a 20 × 40 mm cortical bone flap proximal to the slot entrance (tectoplasty) [16,22,23]. The bone graft was inserted into the slot with its concave cortical surface oriented toward the joint capsule and was carefully advanced while elevating the bone flap [14]. Finally, cancellous bone was packed between the graft and the elevated cortical flap to stabilize the construct.

### 2.3. Data Analysis

We conducted a detailed analysis of changes in acetabular bony coverage and bone graft characteristics following modified Spitzy shelf acetabuloplasty using 3D models reconstructed from preoperative and postoperative CT images. First, the bone graft was evaluated in terms of its dimensions, insertion angle, and placement position based on the reconstructed 3D models. Second, acetabular bony coverage was assessed using the ASA [21], measured from radial sections centered on the femoral head to capture circumferential 3D changes. The LCEA was also measured at multiple locations in coronal and sagittal sections [24]. Third, as a supplementary analysis, conventional 2D radiographic parameters, including the LCEA [24], Sharp angle [25], and AHI [26], were measured on anteroposterior pelvic radiographs to allow comparison with the 3D evaluation. Finally, clinical outcomes were assessed using the Japanese Orthopaedic Association (JOA) score [27] and the Harris Hip Score (HHS) [28].

### 2.4. Image Analysis Method

The workflow for 3D model construction from CT images and registration used for 3D evaluation is described below. First, preoperative and postoperative CT images were exported as axial slices with a slice thickness of 1.5 mm in Digital Imaging and Communications in Medicine format. The image datasets were imported into 3D Slicer [29], an open-source 3D modeling software, for segmentation. The region of interest was defined from the pelvis to the proximal femoral shaft. Bone structures were automatically extracted using a Hounsfield Unit (HU) threshold of ≥200, and original-scale 3D models were generated. Pelvic and femoral models were saved separately in stereolithography format.

In this study, “N points registration” refers to a landmark-based point-to-point registration method implemented in the software, in which anatomically stable pelvic landmarks were manually selected to achieve initial coarse alignment prior to surface-based global registration. “Global registration” was performed using a surface-based registration algorithm with a fixed parameter setting (10 iterations, 15% subsampling), and the average distance error after convergence was used as an internal indicator to confirm sufficient alignment for comparative three-dimensional analysis.

**Figure 2:**
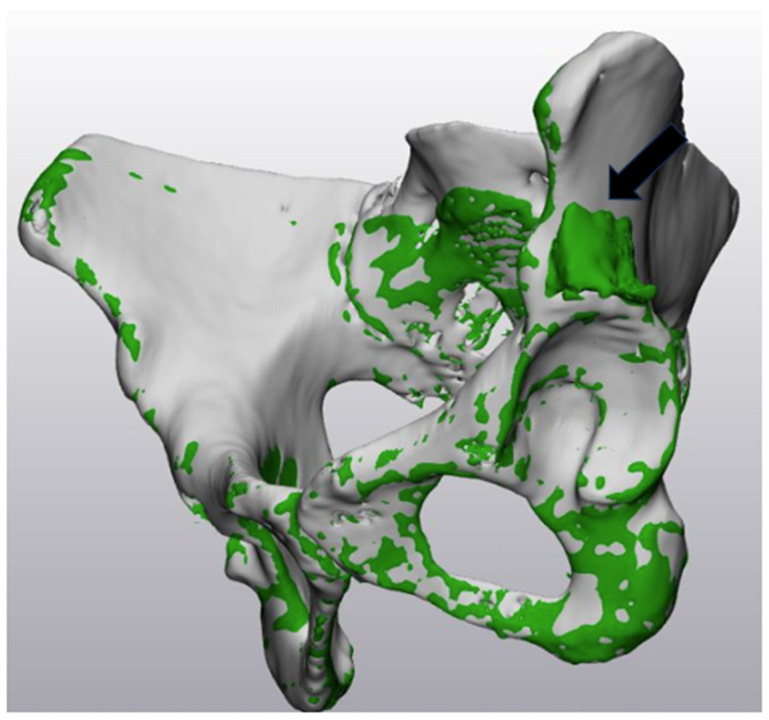
Registration between pre- and post-operative 3D pelvic models. This shows the postoperative 3D pelvic model (green) aligned with the preoperative 3D pelvic model (gray). Proper superimposition has visualized the difference between the two models, revealing the area where bone grafting was performed during surgery (the green grid area indicated by the black arrow).

This process enabled integration of the preoperative and postoperative pelvic models in a common coordinate system, which is essential for the precise evaluation of the 3D positional relationship of the bone graft. The postoperative pelvic model was translated and rotated to match the coordinate system of the preoperative model. The preoperative and postoperative pelvic 3D models were color-coded to visualize the position and morphology of the bone graft (Figure 2).

This approach enabled measurement of the bone graft major axis length in the coronal and sagittal planes, angular assessment of the anteroposterior coverage range relative to the acetabulum, and calculation of insertion angles, thereby providing the basis for quantitative 3D evaluation in this study.

### 2.5. Size and Position of Bone Graft

**Figure 3:**
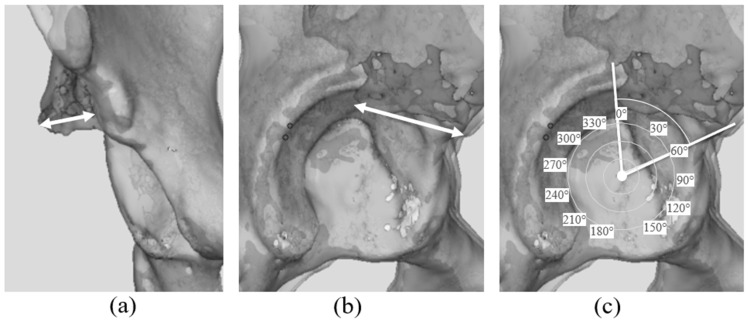
Evaluation of bone graft placement position and dimension. (**a**) Measurement of the distance of the bone graft size (mediolateral length) (white arrow: the linear distance from the outer to inner margin of the bone graft). (**b**) Measurement of the distance of the bone graft size (anteroposterior length) (white arrow: the linear distance from the anterior to posterior margin of the bone graft). (**c**) Evaluation of the coverage range of the bone graft on the acetabular rim (white line: angle representing the coverage range of the bone graft).

The size of the bone graft was quantified in both the sagittal and coronal planes using the registered 3D models. The linear distances between the medial and lateral margins of the grafted bone in the coronal plane and the linear distance between the anterior and posterior margins of the grafted bone in the sagittal plane, was measured. was recorded (Figure 3a,b).

To determine which regions of the acetabulum were augmented by the bone graft, the angular positions of the anterior and posterior graft margins in the sagittal plane were identified using the previously described angular method. The coverage angle provided by the graft was then calculated as the angular span between these two margins (Figure 3c).

### 2.6. ASA Evaluation

Acetabular bony coverage was assessed using the ASA [21] to quantify circumferential changes before and after surgery. The ASA is an index that evaluates acetabular coverage circumferentially by measuring radial sections taken every 15°. Preoperative CT data and postoperative 3D models were imported into 3D Slicer for analysis.

**Figure 4:**
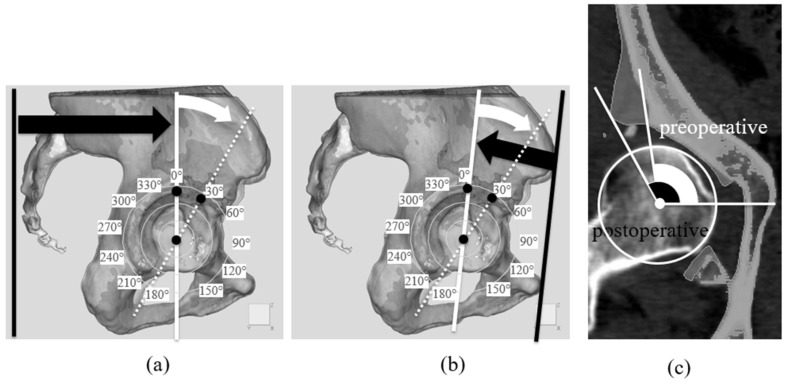
Evaluation method for the Acetabular Sector Angle (ASA). (**a**) The method for creating radial sections is shown. Using the preoperative and postoperative 3D models, radial sections were created by rotating 15° around an axis passing through the center of the femoral head. The position of the acetabular rim apex was defined as 0°, with the opposite acetabular rim at 180°, anterior at 90°, and posterior at 270°. Angles were defined clockwise for the right hip and counterclockwise for the left hip to unify the notation for both hips. The black line indicates the functional pelvic plane (FPP), defined using the inferior boundary of the axial CT image and the CT scanner coordinate system, which provides a standardized pelvic reference orientation for three-dimensional analysis, whereas the white line represents the reconstructed reference plane shifted to the 0° position to make radial section planes (Black arrow indicates the shift direction), and the white dotted line shows the plane for evaluation at the defined 30° position (white arrow indicates the rotational direction) as one example. (**b**) Using a similar method, the anterior pelvic plane (APP) was set as another reference plane. The black line represents the reference plane, whereas the white line represents the plane moved to 0°, and the dotted line represents the plane at the defined 30° position for evaluation. The black and white arrows serve the same purpose as in (**a**). (**c**) The ASA measurement method is shown. In the radial section, the angle between the center of the femoral head and the acetabular rim was defined as the ASA, and comparisons were made between preoperative (white) and postoperative (black) ASA.

To provide a standardized pelvic reference system for three-dimensional analysis, we defined the reference plane as the functional pelvic plane (FPP) based on the CT scanner coordinate system [30]. As illustrated in Figure 4a, the vertical black line represents the inferior boundary of the axial CT field (i.e., the table-side direction), which was used to establish the pelvic reference orientation corresponding to the FPP. In this reference system, the anteroposterior axis was defined as the axis perpendicular to the CT table. This FPP-based coordinate system was then used to generate radial sections for subsequent acetabular sector angle (ASA) evaluation.

For acetabular rim position notation, we adopted a definition in which the acetabular rim apex position was 0°, the opposite acetabular rim was 180° when the reference plane was moved to this position, anterior was 90°, and posterior was 270°. Therefore, angles were set clockwise for the right hip and counterclockwise for the left hip to unify the notation of measurement positions for both hips (Figure 4a). Furthermore, the ASA was measured using the anterior pelvic plane (APP) as the reference [31], applying the same method for radial sectioning (Figure 4b). In each section, the angle formed by lines from the femoral head center to the acetabular rim was defined as the ASA (Figure 4c). ASA values were plotted on radar charts to visualize acetabular coverage before and after surgery [32]. This multivariate representation [33] enabled the evaluation of circumferential coverage, with inward shifts toward the chart center indicating reduced bony coverage.

### 2.7. 3D-Derived LCEA and Bone Graft Insertion Angle

Additionally, two previously unevaluated indicators of acetabular coverage—the three-dimensionally derived lateral center-edge angle (3D-derived LCEA) and the bone graft insertion angle—were established and assessed using an STL viewer (MiniMagics ver. 23.5, Materialise HQ, Leuven, Belgium), which allowed simultaneous visualization of the registered preoperative and postoperative pelvic three-dimensional models (Figure 2).

**Figure 5:**
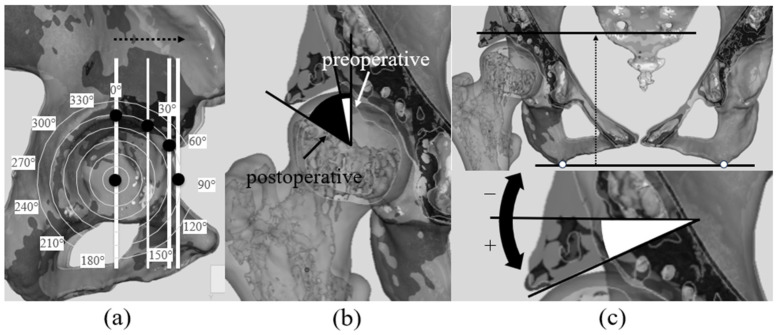
Evaluation of the 3D-derived lateral center edge angle (LCEA) and bone graft insertion angle. (**a**) Coronal plane at positions 0°, 30°, 60°, and 90° were created with white lines, showing the positions for LCEA measurement at each section. (**b**) In each coronal plane, the 3D-derived LCEA was measured as the angle between the line connecting the femoral head center and the lateral margin of the acetabulum and the vertical line (white). (**c**) The method for measuring the bone graft insertion angle is shown. The line connecting the ischial tuberosities was set as the reference axis, and the angle between this reference axis and the long axis of the bone graft was measured as the “insertion angle.” For the right hip, counterclockwise was considered positive, whereas clockwise was considered negative; for the left hip, this measurement was reversed to unify the measurements for both hips.

For calculation of the 3D-derived LCEA, coronal planes were first generated at four predefined angular positions relative to the femoral head center (0°, 30°, 60°, and 90°), as illustrated in Figure 5a. On each coronal plane, the 3D-derived LCEA was defined as the angle between a cranial–caudal line passing through the femoral head center and a line connecting the femoral head center to the lateral margin of the acetabulum (Figure 5b). It should be explicitly noted that these 3D-derived LCEA values are not directly comparable to conventional radiographic LCEA measurements, as they are obtained from three-dimensional CT-based models rather than standardized two-dimensional radiographs.

All reference directions used for these measurements were defined with respect to the functional pelvic plane (FPP). In this reference system, the cranial–caudal direction corresponds to the axis orthogonal to the FPP, whereas the horizontal direction lies within the plane of the FPP.

The bone graft insertion angle was defined on each coronal plane as the angle between the horizontal reference line and the line representing the inferior surface of the transplanted bone (Figure 5c). For the right hip, counterclockwise rotation from the reference axis was defined as positive, whereas clockwise rotation was defined as negative; this convention was reversed for the left hip to unify the measurement orientation between sides.

### 2.8. Clinical and Radiological Evaluation

Radiological evaluation included measurement of the LCEA [24], Sharp angle [25], AHI [26], and assessment of OA grade using the modified Tönnis classification [34]. All measurements were conducted on anteroposterior pelvic radiographs obtained in the supine position. The LCEA was defined by Wiberg and refers to the angle between the line connecting the outer acetabular edge and the femoral head center and the vertical line [24]. The Sharp angle was defined by Sharp and refers to the angle between the line connecting bilateral teardrops and the line to the outer acetabular edge [25]. The AHI was defined by Heyman and Herndon and is an index that evaluates the coverage rate of the femoral head within the acetabulum [26]. OA was evaluated using the modified Tönnis classification [33], with radiological OA progression defined as joint space narrowing to <2 mm. The joint space was defined as the measurement taken on a supine pelvic anteroposterior radiograph at the narrowest point of the weight-bearing region along a vertical line passing through the center of the femoral head [16]. Clinical outcomes were assessed using the JOA score [27], HHS [28], and hip range of motion (ROM).

### 2.9. Statistical Analysis

All statistical analyses were performed using EZR (Saitama Medical Center, Jichi Medical University, Saitama, Japan), a graphical user interface for R 2.13.0 (R Foundation for Statistical Computing, Vienna, Austria). EZR is a modified version of R Commander (version 1.64) designed to add statistical functions frequently used in biostatistics [35]. For the differences between pre- and postoperative situations in ASA [36], 3D-derived LCEA [24], to avoid inflation of type I error due to multiple comparisons across angular positions, angle-specific hypothesis testing was not performed. Instead, repeated measures ANOVA was applied with angular position treated as a within-subject factor to evaluate overall circumferential changes in acetabular coverage. For the differences between pre-and postoperative situations in LCEA, Sharp angle [25], Acetabular Hip Index (AHI) [26], Japanese Orthopaedic Association (JOA) score [27], Harris Hip Score (HHS) [28], and Range of Motion (ROM) were investigated with Wilcoxon signed-rank test. A *p*-value < 0.05 was considered statistically significant. All data are presented as means ± standard deviations.

## 3. Results

In this study, 3D models reconstructed from preoperative and postoperative CT images were used for quantitative analysis. By registering the preoperative and postoperative pelvic models, spatial alignment was achieved, which allowed direct comparison within the same coordinate system. This method enabled precise assessment of bone graft position and orientation and evaluation of changes in bony coverage. Furthermore, clear visualization of bone graft contours enabled reproducible evaluation of parameters, including bone graft size, placement range, and insertion angle.

The measurements using 3D models revealed that the mean bone graft dimensions were 26.3 ± 3.0 mm (anteroposterior length) and 12.7 ± 2.7 mm (mediolateral length). Relative to the acetabulum, bone graft placement was 49.5° ± 10.1° anteriorly and 0.0° ± 6.2° posteriorly, resulting in a mean coverage span of 49.5° ± 9.1°.

**Figure 6:**
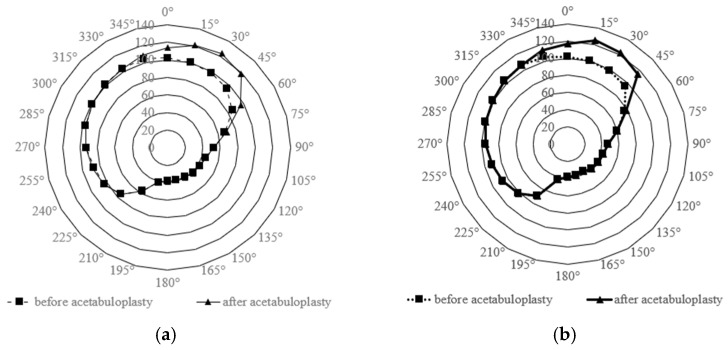
Ci. umferential changes in acetabular coverage evaluated by the Acetabular Sector Angle (ASA). Radar charts illustrate preoperative (square markers) and postoperative (triangular markers) ASA values measured at 15° intervals, using (**a**) the reconstructed reference plane and (**b**) the anterior pelvic plane (APP). ASA represents the angular coverage of the femoral head by the acetabulum in each radial section, allowing visualization of region-specific changes in three-dimensional acetabular morphology.

ASA values in the horizontal plane and APP were plotted using radar charts (Figure 6a,b). In both reference planes, postoperative ASA increased in the sector of 345° to 60°. In both planes, the ASA exhibited significant increases (Repeated Measure ANOVA, *p* < 0.05).

**Figure 7:**
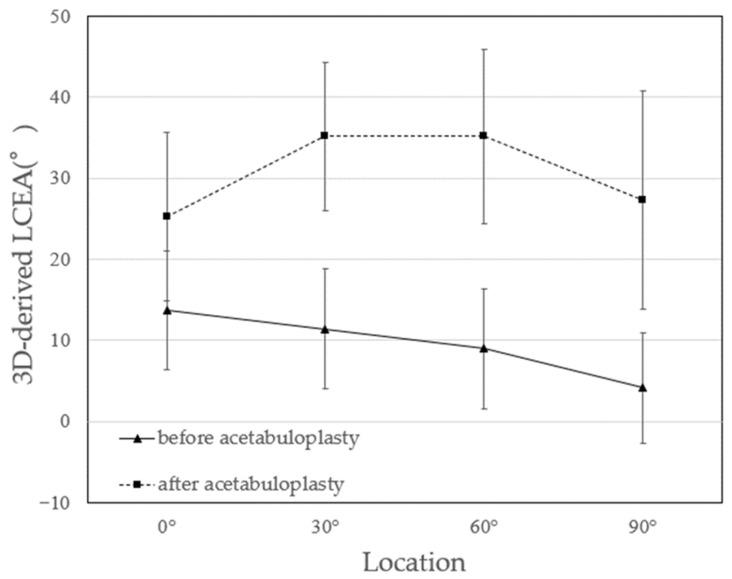
Three-dimensionally derived lateral center-edge angle (LCEA) measured across acetabular regions. LCEA values were assessed on coronal sections rotated around the femoral head center, enabling spatial evaluation of regional changes in acetabular coverage before and after surgery.

LCEA measurements in coronal sections increased significantly at all positions (0°, 30°, 60°, and 90°) (*p* < 0.05) (Figure 7). Preoperatively, the LCEA decreased toward the anterior direction, whereas postoperative measurements demonstrated high angles at 30° and 60°. Regarding bone graft insertion angle, the mean values were positive at all positions, demonstrating that the bone grafts were inclined along the femoral head (Table 3).

On plain pelvic radiographs, the LCEA improved significantly from 9.3° ± 4.5° preoperatively to 35.9° ± 9.0° postoperatively. The Sharp angle decreased from 50.1° ± 5.6° to 39.6° ± 3.8°, and the AHI increased from 59.2% ± 10.9% to 89.9% ± 7.0% (all *p* < 0.05) (Table 4).

The mean JOA score increased significantly from 68.2 preoperatively to 92.4 at the final follow-up (*p* < 0.05). Similarly, the HHS increased from 68.9 to 92.4 (*p* < 0.05). ROM exhibited no significant differences between preoperative and postoperative evaluations. Corresponding *p*-values for each parameter are provided in Table 5.

## 4. Discussion

This study evaluated 3D changes in acetabular bony coverage before and after modified shelf operation and identified morphological changes not detected by 2D assessments. The novelty lies in the fact that we aligned two 3D models created from pre- and post-operative CT scans and evaluated the degree of improvement in coverage not only by measuring it (as 3D derived LCEA) with conventional LCEA but also by quantifying and assessing it three-dimensionally as ASA. Our results confirmed significant alterations in the anterosuperior region of the acetabulum. Radiological evaluation, which relies on measuring the LCEA in coronal sections, is limited to evaluating lateral coverage and fails to consider the 3D form of acetabular morphology. By reconstructing 3D models from preoperative and postoperative CT images, we quantitatively analyzed these spatial changes, including bone graft position. This approach exhibited significant changes in anterosuperior acetabular coverage and highlighted the superiority of 3D evaluation in capturing these changes. These findings underscore that 3D evaluation is valuable for accurately evaluating the morphological effects of modified shelf acetabuloplasty beyond the constraints of 2D evaluation.

This study applied the specific registration steps to superimpose pelvic 3D models reconstructed from preoperative and postoperative CT images, enabling evaluation of acetabular morphology within a common coordinate system. While two-dimensional radiographic assessments can measure lateral coverage, they fail to capture the three-dimensional orientation of the acetabulum. Although previous studies have used CT to assess outcomes after pelvic osteotomy [37], most comparisons were limited to different coordinate systems, such as pre- versus postoperative hips or contralateral sides. The registration-based approach employed in this study, which has been widely applied in other anatomical and clinical fields including growth-related morphological assessment [38], dental movement analysis [39], surgical outcome evaluation [40], and intraoperative procedure management [41], allowed reproducible three-dimensional evaluation of bone graft position and orientation. This approach provided insights into the morphological effects of modified Spitzy shelf acetabuloplasty that could not be obtained using conventional two-dimensional evaluation and may contribute to the standardization of surgical techniques and prediction of postoperative outcomes.

3D evaluation of the size and placement of bone grafts was conducted following modified Spitzy shelf acetabuloplasty. Radiographic evaluations are limited in their ability to measure depth accurately; therefore, detailed evaluation of bone grafts has been limited. Reconstruction of CT images into 3D models enabled the analysis of bone graft size and position from multiple planes. The results revealed that the average bone graft size was 26.3 ± 3.0 mm (anteroposterior length) and 12.7 ± 2.7 mm (mediolateral length). The anterior and posterior extents of the graft corresponded to 49.5° ± 10.1° and 0.0° ± 6.2°, respectively, leading to a coverage range of 49.5° ± 9.1°. Furthermore, insertion angles were positive in all cases, indicating a caudal inclination of 5–20°. These findings corresponded closely with the surgical technique, considering the typical graft size harvested from the iliac crest (approximately 30 mm × 35 mm) and insertion depth (approximately 10 mm). The maintained graft dimensions on postoperative CT images suggest adequate graft incorporation, although detailed evaluation of bone union and resorption was not performed in this study. One previous study reported biological changes in grafted bone fragments [42]. Summers et al. reported bone resorption in 6 of 27 hips after shelf acetabuloplasty, attributing this to a high graft placement that led to insufficient loading [42]. In contrast, our study evaluated bone grafts using CT images obtained after an appropriate postoperative interval, allowing the assessment of graft integrity and the effects of loading. Our findings suggested that appropriately placed grafts were subjected to sufficient loading and remained intact without resorption. Furthermore, bone grafts inserted along the joint capsule were caudally inclined in the coronal plane, oriented to provide anterosuperior coverage of the femoral head. The use of 3D models reconstructed from CT images enables precise assessment of bone graft characteristics.

Although Hounsfield Unit (HU) information was inherently available in the CT datasets, we intentionally did not perform quantitative HU comparisons between the grafted bone and the native ilium, as simple mean HU values without density phantom calibration may lead to oversimplified or potentially misleading interpretations of graft maturation and biological incorporation.

In this study, acetabular bony coverage was evaluated using the ASA. The acetabulum was divided into 15° segments, and changes in bony coverage were analyzed before and after surgery. Significant postoperative increases in bony coverage were observed using both two reference planes. Radar charts were employed to facilitate visualization because they are well-suited for representing multivariate data [32] and enable intuitive comparison of preoperative and postoperative changes. Prior studies using cross-sectional imaging with CT reconstruction to assess acetabular morphology were limited [43]. When derived from CT data, the ASA enables quantitative evaluation of acetabular coverage anterior and posterior to the femoral head [44]. ASA assessment has been used to classify acetabular dysplasia into three deficiency patterns: anterolateral, posterolateral, and global [45]. In Japan, the incidence rates of 26% anterolateral, 20% posterolateral, and 54% global deficiency have been reported [23]. In the present study, the postoperative increase in the ASA was most pronounced in the anterosuperior region, indicating that modified Spitzy shelf acetabuloplasty can effectively compensate for anterolateral deficiency. This indicates that 3D ASA evaluation is not only for characterizing deficiency patterns but also for determining how surgical techniques restore coverage in specific acetabular regions. Although our analyses were conducted at the acetabular center, other studies have evaluated the ASA at different heights to identify deficient areas more comprehensively [46]. The ASA was measured using both the horizontal plane and the APP-based plane. Our findings demonstrated that the extent of postoperative improvement varied depending on the reference plane, highlighting that plane selection influences ASA results, a novel observation warranting further study. Our findings confirmed that modified Spitzy shelf acetabuloplasty primarily enhances anterosuperior acetabular coverage, highlighting the importance of this region in hip joint stability.

For the 3D-derived LCEA evaluation, 3D pelvic models were employed to measure changes in bony coverage across multiple positions in supine coronal sections. Based on the ASA results, the assessment focused on the anterosuperior acetabulum, where the most pronounced changes were anticipated. By integrating ASA and LCEA evaluations, we captured bony coverage changes from complementary perspectives and exhibited the 3D effects of modified Spitzy shelf acetabuloplasty. In this study, evaluating the 3D-derived LCEA in multiple planes enabled the identification of the locations exhibiting the greatest postoperative change. Preoperatively, peak LCEA values clustered near 0°, whereas, postoperatively, they shifted toward 30° and 60°, suggesting that bony coverage improved predominantly in the anterosuperior region. This supports the interpretation that previous reports may have assessed bony coverage at different locations—at the acetabular apex preoperatively and more anterosuperiorly after surgery. By eliminating uncertainties inherent in the conventional LCEA measurements, 3D model evaluation provided a more accurate and spatially comprehensive understanding of acetabular coverage changes and allowed us to capture the morphological effects of shelf acetabuloplasty from multiple perspectives. Other 2D evaluations, including the Sharp angle and AHI, as well as clinical assessments using the HHS and JOA scores, were performed. All parameters exhibited significant postoperative improvement compared with preoperative values.

The purpose of the present statistical approach was not to determine significance at each individual 15-degree interval, but to assess whether a global change in the circumferential coverage pattern occurred after surgery. Accordingly, Figure 6 is intended as a visualization tool to facilitate intuitive interpretation of three-dimensional morphological changes, rather than as a substitute for angle-specific statistical comparisons.

This study has several limitations that should be acknowledged. First, because of the small sample size, caution is required when generalizing the findings. Since the number of men is also small, great care must be taken when applying the results to men. If the number had been larger, the relationship between clinical outcomes and improved coverage might have become clear, but that was not possible. Second, the mean follow-up period of 61.2 months (range, 12–120 months) was relatively short, and long-term clinical outcomes and remodeling of the grafted bone were not fully assessed. Long-term follow-up will be required to evaluate postoperative bone changes and functional prognosis. Third, although the size and position of the bone graft were assessed in this study, the degree of bone union and the presence or absence of resorption were not examined in detail. CT-based assessment of bone union will be required to clarify the long-term fate of bone grafts. Fourth, intra- or inter-observer reliability analysis was not performed. However, according to past literatures [47,48], both have already been reported to have high reliability. Finally, analyses using 3D models have inherent limitations. The accuracy of the present approach depends on the precision of preoperative and postoperative registration and the resolution of the analysis software and thus may involve some degree of error. The introduction of higher-precision 3D analysis methods remains a task for the future. (e.g., if including a bone mineral density phantom during CT scanning might have allowed for a density evaluation). Further comparative studies involving different surgical techniques and patient backgrounds are expected to elucidate the optimal size and placement of bone grafts in acetabuloplasty and to verify their effectiveness. By determining the optimal size and placement of bone grafts, it may be possible to establish a more ideal biomimetic approach, not only in terms of shape but also function.

## 5. Conclusions

As a pilot study, this work demonstrates that modified Spitzy shelf acetabuloplasty enhances anterosuperior acetabular bony coverage, as evaluated using three-dimensional CT-based pelvic models. By aligning preoperative and postoperative 3D models within a common coordinate system, this preliminary analysis enabled spatial and quantitative assessment of acetabular coverage and bone graft orientation that cannot be achieved with conventional two-dimensional radiographic methods.

Although the present findings are derived from a limited and highly specific patient cohort, this pilot investigation establishes the feasibility and utility of a three-dimensional evaluation framework for assessing postoperative acetabular morphology. The proposed approach may serve as a methodological foundation for future studies involving larger and more diverse populations, with the aim of further clarifying the relationship between three-dimensional acetabular coverage, surgical technique, and clinical outcomes.

## Figures and Tables

**Table 1:** Historical reviews of joint-preserving pelvic surgeries.

Operation	Inventor (Year)	Description
Chiari osteotomy [7]	Chiari (1952)	The acetabulum is osteotomized and shifted laterally to improve femoral head coverage.
Transpositional osteotomy [8]	Nishio (1956)	A part of the lateral acetabular wall is osteotomized and translated anterolaterally. Quadrilateral surface (QLS) osteotomy is performed via a lateral approach.
Triple osteotomy [9]	Steel (1973)	Osteotomies are performed at the ilium, pubis, and ischium, allowing rotational correction of the acetabulum.
Rotational acetabular osteotomy [6]	Ninomiya (1984)	The acetabulum is cut in a spherical fashion and rotated anterolaterally. QLS osteotomy is not performed, using a lateral approach.
Periacetabular osteotomy (PAO) [5]	Ganz (1988)	The acetabulum is rotated in three dimensions by cutting the ilium, pubis, and posterior segment without osteotomy of the ischium. QLS osteotomy is performed via an anterior approach.
Curved periacetabular osteotomy (CPO) [10]	Naito (1995)	A modification of PAO in which the osteotomy is curved to allow rotation. QLS osteotomy is performed via a lateral approach.
Spherical periacetabular osteotomy [11]	Hara (2004)	An improvement of CPO enabling a more natural three-dimensional rotation using spherical osteotomy. QLS osteotomy is not performed; anterior approach used.
Shelf operation [12]	König (1891)	A bone graft is placed on the superior acetabular rim to increase coverage. No osteotomy is performed.

**Table 2:** Patient demographics.

Variable	Data
Number of patients/hips	11/11
Age at surgery, mean (range)	30.2 (16–45) years
Sex (male:female)	1:10
Body mass index, mean (range)	22.7 (16.7–29.1) kg/m^2^
Follow-up duration, mean (range)	61.2 (12–120) months
Preoperative modified Tönnis grade (hips)	Grade 0:8 Grade 1:3

**Table 3:** Insertion angles of the grafted bone fragments at each acetabular location.

Location of Acetabulum	Insertion Angle of Grafted Bone Fragment (°; Mean (SD))
0°	4.2 (16.0)
30°	16.8 (6.3)
60°	15.0 (6.9)
90°	7.6 (8.9)

Values are represented as (mean; standard deviation).

**Table 4:** Radiological evaluations showing changes before and after acetabuloplasty (mean (SD)).

Variables	Preoperation	Last Follow-Up	*p*-Value *
LCEA (°)	9.3 (4.5)	35.9 (9.0)	<0.05
Sharp angle (°)	50.1 (5.6)	39.6 (3.8)	<0.05
AHI (%)	59.2 (10.9)	89.9 (7.0)	<0.05

Values are represented as (mean; standard deviation). (* Wilcoxon Signed-Rank Test).

**Table 5:** Physical parameters during the preoperative and last follow-up periods (mean; SD).

Variable	Preoperative	Last Follow-Up	*p*-Value *
Japanese Orthopaedic Association Hip score	68.2 (9.2)	92.4 (8.4)	<0.05
Harris hip score	68.9 (7.0)	92.4 (8.3)	<0.05
**Range of motion (ROM)**			
Flexion	115.5 (15.7)	115.0 (15.6)	0.936
Extension	2.7 (4.7)	1.8 (6.0)	0.588
External rotation	36.4 (15.0)	35.5 (15.0)	0.821
Internal rotation	41.8 (12.5)	39.1 (15.8)	0.573
Abduction	39.1 (7.0)	39.5 (5.7)	0.871
Adduction	18.6 (7.1)	20.9 (8.3)	0.340

(* Wilcoxon Signed-Rank Test).

## Data Availability

The datasets presented in this article are not readily available because the data are part of an ongoing study. Requests to access the datasets should be directed to the Corresponding Author.

## Abbreviations

The following abbreviations are used in this manuscript:

AHI	Acetabular Head Index
APP	Anterior pelvic plane
ASA	Acetabular Sector Angle
CT	Computed tomography
HHS	Harris Hip Score
HU	Hounsfield Unit
JOA	Japanese Orthopaedic Association
LCEA	Lateral Center Edge Angle
ROM	Range of motion
